# Microbe-Driven Genotoxicity in Gastrointestinal Carcinogenesis

**DOI:** 10.3390/ijms21207439

**Published:** 2020-10-09

**Authors:** Kimberly Hartl, Michael Sigal

**Affiliations:** 1 Medical Department, Division of Gastroenterology and Hepatology, Charité-Universtitätsmedizin Berlin, 10117 Berlin, Germany; kimberly.hartl@charite.de; 2 Berlin Institute for Medical Systems Biology, Max Delbrück Center for Molecular Medicine, 10115 Berlin, Germany

**Keywords:** microbe-epithelial interaction, gastrointestinal tract, carcinogenesis, epithelial barrier, barrier dysfunction, microbiota, pathogens, pathobionts

## Abstract

The intestinal epithelium serves as a barrier to discriminate the outside from the inside and is in constant exchange with the luminal contents, including nutrients and the microbiota. Pathogens have evolved mechanisms to overcome the multiple ways of defense in the mucosa, while several members of the microbiota can exhibit pathogenic features once the healthy barrier integrity of the epithelium is disrupted. This not only leads to symptoms accompanying the acute infection but may also contribute to long-term injuries such as genomic instability, which is linked to mutations and cancer. While for *Helicobacter pylori* a link between infection and cancer is well established, many other bacteria and their virulence factors have only recently been linked to gastrointestinal malignancies through epidemiological as well as mechanistic studies. This review will focus on those pathogens and members of the microbiota that have been linked to genotoxicity in the context of gastric or colorectal cancer. We will address the mechanisms by which such bacteria establish contact with the gastrointestinal epithelium—either via an existing breach in the barrier or via their own virulence factors as well as the mechanisms by which they interfere with host genomic integrity.

## 1. Introduction

**Figure 1:**
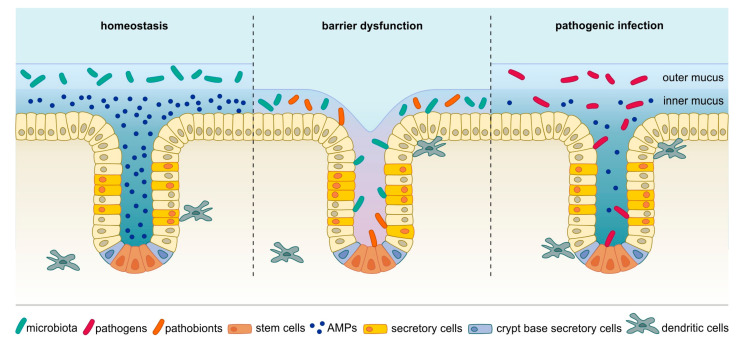
In homeostatic conditions (left), the microbiota resides exclusively in the outer mucus layer. When the barrier is disrupted, for example, through injury, impaired mucus production or chronic inflammation (middle), bacteria, including members of the microbiota and pathobionts, can establish contact with epithelial cells and even invade crypts and interact with stem cells. In addition, distinct pathogens have evolved mechanisms to breach even an intact mucus barrier to make contact with host cells (right). These bacteria can also penetrate glands and adhere to stem cells. AMPs: antimicrobial peptides.

The human gastrointestinal (GI) epithelium forms a barrier between the organism and the surrounding environment. While its main function is to absorb nutrients, it simultaneously needs to prevent exposure to and entry of toxic or pathogenic agents, such as pathogenic bacteria and their toxic metabolites. The epithelium’s columnar monolayer has evolved to act as a semi-permeable interface that is able to actively counterbalance bacterial colonization by multiple means such as mucus secretion, production of a variety of antimicrobial molecules as well as by controlling the luminal microbiota, which in turn limits expansion of potentially dangerous pathobionts or pathogens (reviewed in Chang and Kao [1]). Thus, the natural microbiota itself represents a line of defense for the epithelium. However, due to the need to closely interact with the environment to allow nutrient absorption, the barrier is relatively fragile, enabling microbes to establish contact with the epithelium when the delicate homeostasis is perturbed (reviewed in Vereecke, et al. [2], see Figure 1). Several pathogens such as *Helicobacter* or *Salmonella* have evolved specific mechanisms to breach the epithelial barrier for colonization and invasion [3,4,5,6] (Figure 1). Moreover, various other factors such as genetic predispositions or inflammatory conditions can alter the barrier and disrupt the balance between the epithelium and the resident gut microbes, allowing direct interaction between the epithelium and the microbiota [5,7,8]. In the context of inflammation, which can change epithelial behavior and proliferation, pattern recognition receptors (PRRs) should be mentioned. Members of this class of receptors, such as Toll-like receptors (TLRs), are on one hand needed to maintain homeostasis between microbiota and epithelium [9,10] but can on the other hand also have detrimental effects: activation of TLRs by microbial effectors triggers inflammatory signaling pathways such as nuclear factor ‘kappa-light-chain-enhancer’ of activated B-cells (NF-κB), signal transducer and activator of transcription 3 (STAT3) or nuclear factor of activated T-cells (NFAT), which fuel proliferation, inhibit apoptosis and thus enhance tumor growth [11,12,13,14,15]. Although these signaling can also be critical events in the context of carcinogenesis, this review will focus primarily on the direct DNA damage caused by several bacterial species.

In the healthy condition, the microbiota is kept in a tightly controlled homeostasis in order to prevent bacterial penetration and systemic infection. The epithelial monolayer carries efficient defense mechanisms that allow it to act as a barrier between the bacteria-containing lumen and the host organism itself. In addition to the physical border established by the mucus layer covering the epithelial cell surface, there is also a chemical border generated through active secretion of antimicrobial substances, such as antimicrobial peptides (AMPs), which either kill bacteria directly or interfere with their metabolism. Recognition of pathogens by immune sensors allows for a specifically targeted host response, keeping the normal microbiota intact but destroying the intruder [16]. It is now recognized that in certain conditions such as Crohn’s disease the microbiota composition is altered, which might be due to microbial competition, secreted bacteriotoxins or host-derived bactericidal defense mechanisms [17]. This, together with impaired mucus production, paves the way not only for pathogens but also for commensals to come in contact with the epithelium and even infiltrate the crypts—a process that is not observed under healthy conditions [18].

While these events have been linked to various infectious and inflammatory disorders, there is an increasing interest in the role of bacteria in development of gastrointestinal malignancies [19,20,21,22,23]. Indeed, the GI tract epithelium is a site particularly prone to malignant disease: stomach and colorectal cancer (CRC) alone accounted for about 3 million new cancer cases and 1.5 million deaths in 2018, making them the second and third deadliest among all cancers, respectively [24]. While formerly it was believed that these cancer types, particularly CRC, are mainly driven by genetic predisposition and stochastic mutations, there is now increasing evidence that bacteria can contribute to and even actively drive carcinogenesis by changing the microenvironment or cell signaling or by directly altering host DNA integrity.

**Figure 2:**
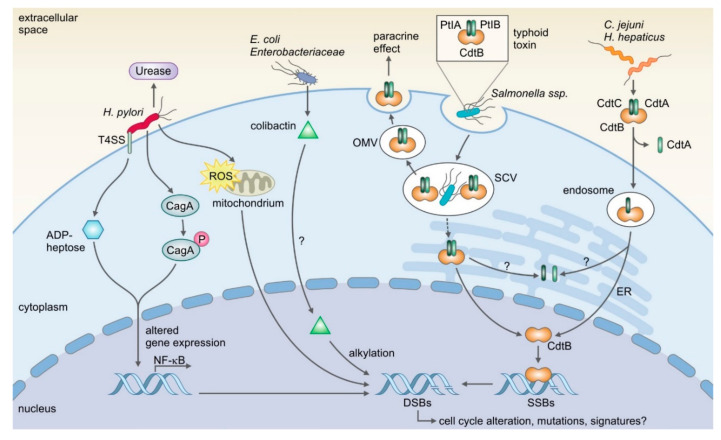
Overview of microbes and their specific virulence factors that can induce DNA damage. H. pylori’s type IV secretion system (T4SS) is known to be crucial for induction of DNA damage. The injected effectors ADP-heptose and CagA alter gene expression, which leads to the expression of NF-κB target genes and DNA damage. Additionally, production of reactive oxygen species (ROS) is believed to be a cause for DNA damage. E. coli and other Enterobacteriaceae produce the genotoxin colibactin, which has been shown to induce DNA double-strand breaks (DSBs) via alkylation. However, it is not clear yet how the genotoxin is delivered to the nucleus. In contrast to this, the shuttling process of typhoid toxin and the related cytolethal distending toxin (CDT), which share the catalytic subunit CdtB, have been resolved for several species. Typhoid toxin is produced by the typhoidal Salmonella strains Salmonella enterica Paratyphi A and Typhi, while CDT is expressed by several species such as Campylobacter jejuni and Helicobacter hepaticus. While the trafficking is different for the holotoxins, the mutual CdtB subunit, a homolog to DNase I, is translocated into the nucleus where it induces single-strand breaks (SSBs) that can ultimately become DSBs. It is still under investigation how the induced DSBs can lead to mutations and cancer. For colibactin is has recently been shown that it causes a mutational signature, which could help reveal the underlying mechanisms. PtlA/B: Pertussis toxin transport protein or Pertussis toxin liberation A/B; CdtA/B/C: cytolethal distending toxin subunit A/B/C; OMV: outer membrane vesicles; SCV: Salmonella containing vacuole; ER: endoplasmic reticulum.

While the causal link between cancer development and viral infection is widely accepted, such a link is more difficult to establish for bacteria, which are not known to integrate genetic material into the host genome. Although epidemiological as well as in vitro evidence exists for a carcinogenic effect of some bacterial infections, the mechanisms by which bacteria trigger carcinogenesis are not yet fully understood and under active investigation. One common feature in this context is that direct interaction of bacteria with host cells is required for DNA injury. Some pathogenic bacteria possess the ability to counteract the epithelial defense mechanisms to make contact with host cells, leading to DNA damage. Although other pro-carcinogenic mechanisms, such as inflammatory responses or action of miRNAs, have been proposed for distinct bacteria such as *Bacteroides fragilis* or *Fusobacterium nucleatum*, a common feature of several bacterial species that are epidemiologically linked to cancer is that they cause DNA damage. This can occur indirectly, via increased levels of reactive oxygen species generated by the host’s immune defense, especially upon chronic infection. However, it can also occur directly and bacteria that can cause direct DNA damage and are connected with malignant diseases will be in the focus of this review. Here, we describe how pathogenic and pathobiontic bacteria contribute to DNA damage and epithelial carcinogenesis. Specific genotoxins and other bacterial virulence factors that are epidemiologically and mechanistically linked to DNA damage and carcinogenesis have recently been described and characterized (see Figure 2). Various genera possess genotoxins, including the cytolethal distending toxin (CDT), which causes DNA double-strand breaks (DSBs) [25]. Others alter the host DNA damage response, potentially resulting in impaired repair, mutations and cancer (reviewed in Chumduri, et al. [26]). Such genotoxins and other factors can be transferred from the bacteria to the host cell via secretion systems, for example, outer membrane vesicles or type IV secretion systems (T4SS). In all of these cases, direct interaction with the cells, for example, adherence, ingestion or at least close proximity is necessary for the microbes to alter cell signaling.

Another important aspect is the heterogeneity and different life-spans of epithelial cell types in the gut: most cells are differentiated and short-lived, while the stem cells in the crypt base are long-lived, indicating that damage to these cells could be a necessary event for maintenance and fixation of genomic lesions.

Considering these aspects, we propose that in order to contribute to gastrointestinal cancer onset, bacteria must meet two criteria: Firstly, they must be able to establish direct contact with the epithelium either via active mechanisms or through an independent event leading to a disruption of the epithelial barrier, for example, previous damage. Secondly, they must possess molecular virulence factors that cause DNA damage and mutations. For such mutations to persist in the tissue, it is most likely required that long-lived cells, such as stem cells, are targeted by genotoxic events. 

This review recapitulates the current understanding of the interaction between carcinogenic microbes and the host epithelial cells in the GI tract. We summarize epidemiological insights as well as insights into the mechanisms that link bacteria and their virulence factors to DNA injury and carcinogenesis. Additionally, this review is accompanied by a list of the most relevant reviews of the topic (see Supplementary Materials Table S1).

## 2. The Epithelial Barrier

The epithelial barrier in the GI tract is equipped with several levels of defense [27,28,29] responsible for maintaining a distance between the luminal bacteria and the gut mucosa.

The most prominent line of defense is the mucus. In addition to forming a physical barrier, the mucus contains a variety of proteins with antimicrobial activity, many of which are produced by specialized subpopulations of epithelial cells. In addition, there are also region-specific mechanisms within the gastrointestinal tract that limit bacterial colonization. For example, gastric parietal cells secrete acid, creating a highly acidic and bacteriotoxic environment in the stomach [30,31,32]. In the colon, beta oxidation in enterocytes is crucial for maintenance of an anaerobic luminal environment, which supports microbiota diversity and resistance to colonization with pathogens as well as carcinogenic pathobionts [33,34,35,36].

Another aspect that appears to be important for maintaining homeostasis between the microbiota and the host is the crypt-like structure of the epithelium with long-lived stem cells located in the very base, distant from the lumen. Several defense mechanisms appear to specifically protect these stem cells, such as their location and direct proximity to antimicrobial secretory cells, like the Paneth cells of the small intestine [37,38,39]. In the following section, we will discuss the most prominent features of epithelial self-defense in more detail.

### 2.1. Mucus

Mucus covering the epithelial layer can be found throughout the whole gastrointestinal tract, although its thickness and structure vary between the different sections, from 150 µm in the small intestine to 700 µm in the stomach and colon [40]. While in the stomach and colon the mucus layer is separated into an inner and an outer layer, in the small intestine the separation into layers is less apparent [41]. Under healthy conditions, the adherent inner mucus layer is free from bacteria but the loose outer mucus layer is populated by the microbiota [18] making it the primary site of microbiome-host symbiosis. Together with the complex glycans provided by food digestion, the mucus serves as a nutrient source and attachment site for mucolytic commensals, which in turn provide short-chain fatty acids and other energy sources to the host epithelium and other members of the microbiota [42,43,44,45]. In addition to these probiotic effects, the mucus also contains several antimicrobial factors, including mucins large, highly glycosylated proteins secreted by goblet cells [46,47,48], defensins—cationic peptides with antibiotic action that are produced by epithelial cells [49], other AMPs and a specific proportion of water and ions. Also, secretory IgA and IgG antibodies are mucus components, blocking bacteria from binding to the epithelium [50]. Although mucus composition and secretory cell location differ from organ to organ [51], the whole epithelium of the GI tract possesses mucus as a common feature. Mucins can be divided into two classes: Classic gel-forming mucins (MUC2, MUC5AC, MUC5B and MUC6) that form polymers and constitute the mucus layer and transmembrane mucins (e.g., MUC1, MUC3, MUC12) that are located on the apical side of enterocytes as part of the glycocalyx, the glycoprotein and -lipid covering surrounding the cell membrane [52]. The mucus is a physical barrier and prevents bacteria from moving towards the epithelium by its viscous texture promoted by these polymeric glycoproteins [53]. But it also is a chemical barrier, featuring bactericidal substances like β-defensin 2, which is expressed in several types of epithelia [54,55]. Goblet and Paneth cells are the known cell types of the GI tract responsible for gel-forming mucin production and secretion, for example, mucin 2 (MUC2), which is the most critical mucin in the intestine or MUC5AC, which is expressed in the stomach [18,56]. The importance of the mucus barrier function is illustrated by the fact that mice lacking MUC2 develop spontaneous inflammation of the colon [18,57] and similarly, deficiency in cell-surface mucin MUC1 makes mice more susceptible to severe infection with *H. pylori* or *Campylobacter* species [58,59,60]. In addition, mucins act not only in a protective but also in an antimicrobial manner: MUC6 in the stomach is believed to limit growth of *H. pylori* within the mucus by inhibiting its cell wall biosynthesis [61]. The importance of the mucus is also reflected clinically, as biopsies from ulcerative colitis patients showed that the thickness of the mucus layer is reduced proportionally to the severity of the inflammation and that bacteria were present within the mucus in colitis samples [62]. Besides this, MUC2 expression and the number of goblet cells as well as their secretory capacity is decreased in these patients [63].

### 2.2. Antimicrobial Proteins

AMPs are part of the non-specific innate immune response, either directly killing or inhibiting the growth of microbes, most commonly by targeting the bacterial cell wall [64,65]. Having evolved early during evolution, AMPs can be found in almost all members of flora and fauna [66]. So far, more than 3200 AMPs from six kingdoms have been identified [67]. Although most of them share common features such as small size (12–50 amino acids), positive charge and amphipathicity, gut antimicrobials can be divided into three key families: defensins, cathelicidins and C-type lectins [64,65,68]. Defensins have a very broad spectrum of action, disrupting membranes in many different microbial species regardless of their Gram status and even in fungi and protozoa [69,70,71]. Through electrostatic interaction defensins form pores in the bacterial membrane, disrupting it and ultimately leading to lysis [72]. There are two classes: α-defensins, which are produced by Paneth cells in the small intestine [69] and β-defensins, which are mainly produced in the colonic epithelium [73]. Their expression is also regulated differently: α-defensins are expressed independently from the microbiota while most β-defensins such as BD2 (also known as DEFB4A) require microbial signals for induction [74,75]. Expression of α-defensins is crucial for a functioning host defense against pathogens. For example, mice deficient in MMP7, which is required for α-defensin production, showed increased vulnerability to oral challenge with *Salmonella*, while those overexpressing the human defensin 5 (DEFA4) were more resistant to infection [76,77]. By using these two models it has additionally been shown that α-defensins do not only affect pathogenic bacteria but also take part in regulating the microbiota composition in the intestine [78].

Similar to defensins, cathelicidins disrupt bacterial membranes by forming pores irrespective of Gram status but unlike defensins, they are produced by a variety of cells such as epithelial and immune cells. Despite their direct antimicrobial activity, they also regulate host responses such as proliferation, migration, cytokine release and onset of adaptive immune response [79]. Only one human cathelicidin has been isolated so far—LL-37 or hCAP-18—which is also present in the GI tract [80,81,82,83] and which is thought to be closely related to the murine cathelin-related antimicrobial peptide (CRAMP) [79]. Disruption of its gene, *cnlp,* leads to increased susceptibility to skin infections in mice [84].

The third family consists of C-type lectins (C for calcium-dependent [85]), which are expressed in the small intestine by epithelial cells such as Paneth cells and enterocytes [86,87]. Lectins only act on Gram-positive bacteria, as they recognize bacterial peptidoglycan [88]. However, their mechanism of action is still under investigation [64]. One of the most well-studied murine lectins is REG3γ, which plays a critical role in keeping the inner mucus layer sterile. Knock-out mice suffer from increased colonization by Gram-positive bacteria in the inner mucus layer but not in the luminal content [87].

### 2.3. Protection of Stem Cells

As outlined above, Paneth cells are located near stem cells and are thought to be responsible for keeping the stem cell niche sterile by producing a large variety of AMPs [47,89]. Apart from α-defensins, lectins and cathelecidins, they also produce lysozymes, angiogenin 4, secretory phospholipase A2, lipopolysaccharide-binding protein, collectins, deleted in malignant brain tumors 1 (DMBT1) and histatins [89,90,91,92]. In addition to the presence of Paneth cells, protecting the stem cells from invading microbes is achieved by several means. The stem cell niche in the GI epithelium consists of glands or crypts with stem cells located in the base, the place most distant from the microbiota and its metabolites. Here, stem cells are more protected from potentially intruding microbes and their metabolites than cells at the gland surface. In addition to biogeographical features, it has been observed that the crypt structure and its cellular organization are important for protection of stem cells against potentially harmful metabolites. For instance, it has been demonstrated that the bacterial metabolite butyrate inhibits stem cell proliferation and that its consumption by surface enterocytes, that use butyrate for beta oxidation, prevents the exposure of stem cell to it [93]. As shown by our group, both antimicrobial-producing cells and stem cells in the stomach rely on the same niche factors, such as R-spondin [94], suggesting that antimicrobial protection mechanisms are an integral function of the stem cell compartment that are important for tissue homeostasis. Similarly, in the intestine, Lgr5^+^ stem cells rely on niche factors like Wnt3 or EGF provided by nearby Paneth cells. Conversely, Paneth cells themselves require Wnt ligands for AMPs expression to be induced [95]. Being specifically shielded might be the result of evolutionary pressure to avoid injuries in stem cells: damage in long-lived, proliferative cells is likely to be a critical step in tumor development [96] and thus the crypt structure might be a product of co-evolution between host and pathogen. This allows for replenishment of the potentially damaged surface cells through a constant turnover driven by stem cell proliferation. Of note, in the stomach antimicrobial protein expression is induced upon infection with *H. pylori* in the gland base, suggesting that a full antimicrobial activity requires exposure to bacteria. Indeed, experiments with gnotobiotic mice have shown that the microbiota itself regulates barrier maintenance and is needed to establish Paneth cell function and full barrier functionality [97,98], which might be critical to preserve the host from pathogenic infections.

Despite the multiple levels of defense, some bacteria have evolved mechanisms to disrupt the barrier and to colonize the epithelium-even that of the acidic stomach. Here, *H. pylori* requires the protective mucus layer to colonize and persist, while being able to invade into glands and even colonize stem and progenitor cells. Since direct interaction with epithelial cells also seems to be crucial for other carcinogenic bacteria, an important question is how these microbes manage to gain access to host cells, that is, how do they cross the host defense lines? We will discuss examples of two distinct types of bacteria: pathogens that actively fight host defense mechanisms to establish contact with the epithelium and commensals that can establish this contact only under certain circumstances. In addition, we discuss other bacteria that have been linked to cancer although it is not yet clear how they are able to reach and interact with the epithelium.

## 3. The Model Pathogen: Helicobacter Pylori

*H. pylori* is a spiral-shaped, lophotrichous flagellated, Gram-negative bacterium that colonizes the human stomach [99]. In 1984 Barry Marshall proved that the bacterium is the cause of gastric ulcer formation by administering *H. pylori* to himself [100]. Today, it is well accepted that it induces gastric inflammation, peptic ulcer disease and is a risk factor for gastric cancer development [101,102,103]. Although many bacteria are epidemiologically linked to cancer, *H. pylori* is so far the only one that has been widely accepted as a carcinogenic bacterium and has been classified as g I carcinogen [104]. A cohort study by Judy Parsonnet and colleagues revealed in the early 1990s that *H. pylori* infection is a risk factor for developing gastric adenocarcinoma; 84% of individuals with gastric cancer had a history of *H. pylori* infection. Although infection incidence is rapidly decreasing in the developed world, putatively through improved hygiene, in countries of Southern and Eastern Europe, South America and Asia the prevalence often exceeds 50% [105]. While the percentage of cancer cases attributed to *H. pylori* declined from 2008 to 2018 (6.2% vs. 4.8%), the total number of cases increased (780,000 vs. 810,000) [106,107]. Based on studies that reported a reduction of gastric cancer incidence through *H. pylori* treatment [108], the International Agency for Research on Cancer (IARC) published recommendations for gastric cancer control in 2014, including screening and eradication strategies.

*H. pylori* has evolved persistence mechanisms to overcome the acidic environment of the stomach by producing urease, which buffers the surrounding acid by production of ammonia ions and allows the bacterium to establish a habitable, pH-neutral microenvironment [109,110]. Through rheological studies, it has been discovered that *H. pylori*-induced pH elevation not only favors its survival within the mucus but also enables the bacterium to swim through it by decreasing its viscoelasticity [111]. Thus, *H. pylori* is capable of overcoming this first line of defense to colonize the gastric mucus layer and to move within it. There, in turn, it benefits from the mucus that protects the epithelium from acid [55], while at the same time remaining in close proximity to epithelial cells. While a subpopulation of *H. pylori* is free-swimming in the mucus, some bacteria are able to attach to and colonize the intercellular junctions of epithelial cells. Several molecular adhesins, such as BabA, SabA and HopQ, have been described to be important for the attachment to epithelial cells [112]. Salama and colleagues found that the helical shape of *H. pylori* allows the bacterium not only to swim directedly in the mucus but also to interact with the host and induce inflammation [113]. Although the initial defense response by the epithelium, including expression of β-defensin 3, can kill *H. pylori* effectively, the bacteria are able to overcome this defense by translocating the virulence factor cytotoxin-associated gene A (CagA) into host cells, which inhibits defensin secretion through the tyrosine phosphatase SHP-2 [114,115].

On the tissue level, it was originally shown that *H. pylori* interacts with surface pit cells [116,117]. Three-dimensional confocal microscopy imaging has recently revealed that *H. pylori* is also able to colonize deep in the gastric glands and to interact with stem and progenitor cells in a mouse model of infection as well as in human samples [118], leading to stem cell expansion and gastric pathology [119].

Another factor necessary for colonization is cholesterol-α-glucosyltransferase, which allows *H. pylori* to deplete cholesterol from the host cells, reducing IFN-γ signaling due to disruption of lipid rafts, leading to downregulation of JAK and STAT1 pathways, along with several other cytokines and antimicrobial peptides [120]. This ultimately prevents the infected cells from responding to the inflammatory signals from incoming immune cells and helps *H. pylori* to persist despite ongoing inflammation.

While being beneficial for persistence, *H. pylori’s* direct interaction with epithelial cells, especially with stem cells is thought to be crucial for its carcinogenic effects. Once attached to the epithelium, it can use its T4SS to inject its virulence factors into host cells [115]. In particular, translocation of CagA has a major effect on cellular signaling. Once inside the host cell, CagA is phosphorylated by Src and Abl kinases and can interact with other host proteins, which leads to host cell cytoskeletal rearrangements [115,121,122]. *CagA*^+^ strains are considered to increase the risk for gastric carcinoma and indeed CagA’s oncogenic action has been proven in the mouse model where it causes stomach cancer [123] and in epidemiological studies [124]. Some mechanistic insights suggest that CagA might interfere with the Wnt signaling downstream component β-catenin, releasing it into the cytosol and allowing nuclear accumulation, which results in transcription of proliferation- and differentiation-related genes [125,126]. More recently, another bacterial effector has been found to be critical to activate NF-κB signaling: ADP-heptose which acts as a Pathogen-Associated Molecular Pattern (PAMP) on epithelial cells and is also translocated by the T4SS [127]. NF-κB has been proposed as being upregulated in conditions related to cancer onset and although rarely mutated itself, it plays a central role in a signaling network containing frequently mutated upstream molecules like RAS, EGFR or HER2, resulting in elevated NF-κB signaling and in cross-talk with genes related to ROS production, as well as the p53 pathway and the STAT pathway [128].

In addition to altering signaling pathways, Toller and colleagues found that the T4SS is required for induction of DSBs in host cells by *H. pylori* and that they are less likely to be repaired with ongoing infection [129]. Infection seems to impair a proper DNA damage response, suppressing homologous repair (HR) and enhancing the non-homologous end joining (NHEJ) pathway which leads to more error-prone repair [130]. Later on, the same group could verify that DSBs are induced by host repair mechanisms, namely by nucleotide excision repair (NER), in a T4SS-dependent manner and lead to NF-κB target gene expression, enabling cell survival despite the DNA damage [131].

Taken together there is increasing evidence that the ability of *H. pylori* to interact with epithelial cells and to colonize glands is beneficial for bacterial persistence. The ability of *H. pylori* to manipulate epithelial cell behavior to create a protective niche appears to be causally linked to aberrant cell behavior, DNA damage and risk for carcinogenesis.

## 4. The Enteric Pathogens: *Campylobacter jejuni*, the *Salmonella* Genus and Others

In addition to *H. pylori,* several other pathogens have come into the focus of infection biology research due to their epidemiological connection to distinct types of cancer. This is true for classic pathogens such as *Campylobacter, Vibrio* or *Salmonella* species.

The ability of these pathogens to colonize the gut is well studied, as enteritis is still a common threat that accounts to more than 1.4 million deaths per year [132]. In order to interact with the epithelium, pathogens have to cross the protective mucus layer that is constantly exchanged through flushing and newly produced mucus (reviewed in Frick and Autenrieth [133]). For *Campylobacter*, *Salmonella* and *Vibrio*, penetration is made possible by various virulence factors, such as flagella enabling movement from or towards a stimulus, the spiral shape of the bacterium itself and secreted proteases that simplify movement through the vicious mucus [134,135,136,137]. The molecular mechanisms by which *Campylobacter jejuni* and *Salmonella ssp*. are capable of overcoming host defense mechanisms and establish persistent infection have been reviewed previously (see Burnham and Hendrixson [138], Di Domenico, et al. [139], Ducarmon, et al. [140], Gal-Mor [141], Wagner and Hensel [142], and Young, et al. [143]) so we will only introduce them briefly and focus on exploring possible modes of action through which these microbes may induce cancer.

### 4.1. Salmonella

The Gram-negative, facultative anaerobic species *Salmonella enterica* consists of more than 2500 serovars known to cause food- and water-borne diseases and is ubiquitously present among humans and animals [144]. Infection causes typhoid fever with typical gastrointestinal symptoms like abdominal pain and diarrhea [145] but can also evolve to a potentially fatal systemic disease by dissemination of bacteria to internal organs [146,147]. Although the majority of bacteria ingested are killed by stomach acid, bile or intestinal defensins, if the dose is high enough (only about 1000–10,000 colony forming units) surviving *Salmonella* can colonize the gut lumen or even invade the mucosa and proliferate within epithelial cells [148]. Importantly, from the intestine passing through the liver *Salmonella* can enter the gallbladder [149,150].

Despite the rising incidence in developed countries in North America and Europe [151], salmonellosis and more specifically typhoid fever, is endemic in developing countries such as India or nations in the western parts of South America. Interestingly, the incidence of gall bladder cancer in these regions is much higher (~20-fold) compared to the rest of the world [152,153]. Outbreaks in regions in which typhoid fever is typically not endemic have enabled a clear link to be made to corresponding increases in the rates of gall bladder cancer [154,155].

Different from the serovar commonly used for in vivo research, *S. enterica* Typhimurium, the human-restricted typhoidal serovars Paratyphi A and Typhi, feature the typhoid toxin, a chimeric toxin consisting of the catalytic CdtB subunit of the cytolethal distending toxin (CDT) and two domains of the pertussis toxin, PtlA and PtlB. The typhoidal strains have been linked to an increased risk of developing gall bladder cancer in asymptomatic, chronic carriers, in whom the bacteria reside on biofilms on gall stones in the gall bladder after acute infection. Around 2–5% of typhoid patients do not manage to clear the infection and become chronic carriers [156] which increases the risk for malignant gallbladder disease about 8fold [157]. The typhoid toxin’s CdtB subunit is a homologue of DNase I [158,159]. The CdtB subunit might have been acquired through horizontal gene transfer, as the CDT holotoxin is present in diverse Gram-negative bacteria. The catalytic subunit exhibits DNase activity after internalization and translocation to the nucleus, directly inducing SSBs that lead to DSBs, as well as altering cell cycle progression and the DNA damage response [160,161,162,163], potentially leading to mutations and malignancy (reviewed in Chumduri, Gurumurthy, Zietlow and Meyer [26]). Although it was shown in 2015 that *Salmonella* is able to induce cellular transformation via manipulation of the AKT and MAPK pathway [119], the model used were pre-transformed host cells with a strain lacking the typhoid toxin. So far it is not clear whether and how *Salmonella* could be capable of inducing these primary mutations itself and to what extent such transformation could be caused by the typhoid toxin or other virulence factors.

### 4.2. Campylobacter

*Campylobacter* species are characterized as Gram-negative, motile, microaerophilic, spirally shaped bacteria possessing single flagella at one or both cell poles [164,165]. Although many members of this genus are commensals, others, especially the thermophilic *Campylobacters,* such as *Campylobacter coli* and *Campylobacter jejuni,* have been found to induce gastrointestinal disease [164,166]. In the U.S. an estimated 800,000 cases of campylobacteriosis occur every year and it is believed that *Campylobacter* is responsible for far more food-borne disease cases in the developed world than *Salmonella* [167,168,169,170]. Symptoms are dose-dependent, ranging from mild diarrhea to acute colitis and even sepsis and death [171,172,173,174]. After ingestion, *Campylobacter* colonizes the epithelium of the distal ileum or colon, damaging host cells through adhesion, bacterial toxins or indirectly through the induced inflammatory response [165]. Chemotaxis has been demonstrated to be indispensable for colonization, as are functional flagella for motility within the mucus [137,165]. There is also evidence that the flagella are taking part directly in adhesion through binding mucins [5]. *Campylobacter* is highly motile within the mucus layer, chemoattracted to mucins and binds mucin oligosaccharides [135,175]. Specifically, it has been shown to interact with MUC2 in the intestine, which causes upregulation of several of its virulence genes [176]. The autotransporter protein CapC also plays a role in promoting epithelial adhesion and invasion [177]. While it establishes its niche, *Campylobacter* effectively disrupts tight junctions [178].

*Campylobacter jejuni* is one of the best-known members of this family and is also one of the candidates that has been investigated in connection with CRC, as it expresses CDT, which causes DSBs [179]. There is also accumulating epidemiological evidence for a role of *Campylobacter* in malignant diseases. *C. jejuni* accompanied by *E. coli* is present more often in colorectal cancer lesions than in the adjacent tissue [180,181]. *Campylobacter* also eliminates various taxa from the luminal microbiome once it is established and it has been proposed that this is due to the genotoxic action of CDT [179]. This genotoxic effect not only affects the microbiota but also induces genomic instability in the host, as shown for CDT produced by other Gram-negative bacteria, such as *Helicobacter hepaticus* [182]. It is also known that host cells respond to invading *Campylobacter* by upregulating MUC1 production, which partially protects them from the actions of CDT [59], thus defending not only against the infection but also against potential DNA damage. Although a human clinical isolate of *C. jejuni* has been shown to promote colorectal tumorigenesis in vivo in DSS-treated APC^Min/+^ mice [179], the mode of action is still under investigation and the causal link to CRC remains unproven.

### 4.3. Helicobacter Hepaticus

*Helicobacter hepaticus* is a spirally shaped Gram-negative bacterium slightly smaller than *H. pylori,* has bipolar sheathed flagella and can live in both anaerobic and microaerophilic environments [183]. This pathogen has mainly been investigated in the mouse model, where it causes hepatic diseases such as chronic hepatis [184,185] but may also exhibit pathogenicity in humans as it is detected in patients with hepatobiliary disease [186,187]. Although closely related to *H. pylori* and sharing the ability to produce urease, *H. hepaticus* lacks other key virulence factors of *H. pylori* such as CagA and the three adhesins SabA, AlpA and BabA [183]. By contrast, it expresses the CDT orthologue of *C. jejuni* [183] and may therefore induce mutations through the same mechanism, that is, induction of DSBs and alteration of the DNA damage response. In fact, is has been demonstrated that CDT induces cell cycle arrest at G2/M checkpoint and may therefore also promote persistence of infection [188]. *H. hepaticus* infection causes liver cancer in A/Jct mouse models and correlates with the development of gallbladder polyps and gallbladder cancer in humans [183,184,186,187,189,190]. *H. hepaticus* as well as other *Helicobacter* species are frequently found in cholangiocarcinomas and it has been postulated that they may induce hepatobiliary malignancies [191,192]. In line with this, higher titers of antibodies against *H. hepaticus* were found in patients with gallbladder cancer [189,190]. However, neither the exact route of infection, nor the persistence niche or the causative mechanism of its relation to cancer have yet been proven and research will need to focus on this in order to provide comprehensive explanations for these connections.

In conclusion, pathogenic bacteria of the gastrointestinal tract have the ability to breach the epithelial barrier effectively, leading to symptoms observed during acute infection, such as diarrhea. While the acute effects are well understood, those bacterial species that can partially persist in the host seem to be able to cause long-term effects and contribute to carcinogenesis. It will be important to explore in more detail what types of cells pathogens interact with once they have breached the barrier and how the genomic instability that can be caused by different virulence factors is then propagated in a way that leads to cancer. To provide definitive proof that bacteria causally drive carcinogenesis, the long-term effects of pathogens on the host tissue need to be determined in order to monitor a possible mutational cascade.

## 5. Commensals—To Protect and Serve?

In contrast to invading pathogens, commensals are part of our natural microbiota that lives in symbiosis with us, tolerated by the immune system while in ongoing exchange of nutrients and signals with our organism. However, under certain circumstances, commensals can obtain pathogenic features and harm epithelial cells. Due to the epithelial barrier, commensals usually do not come into contact with the intact epithelium but reside in the outer mucus layer. Although they are not characterized as harmful per se, some of them have been linked to cancer through mechanistic studies and metagenomic analyses (reviewed in Rajagopala, et al. [193]).

Since the microbiota itself can be considered as a line of epithelial defense, it is of interest how commensals, pathobionts and pathogens interact. How the gut microbiota controls pathogens and pathobionts has been reviewed by Kamada, et al. [194]. An imbalance within the microbial population through changes in number and composition can provide a competitive advantage for pathogens or pathobionts to grow. In particular, a high oxygen concentration is a marker for gut dysbiosis as it triggers expansion of pathogens like *Salmonella* ssp. or pathobionts from the *Enterobacteriaceae* family [195,196]. Loss of hypoxia through inflammation and subsequent elimination of probiotic anaerobes disturbs the gut’s natural resistance against colonization by pathogens and can lead to an overgrowth of harmful microbes [196]. Colibactin-producing *E. coli,* which will be discussed in the following section, expand within the microbiota in an AOM/DSS mouse model and this expansion is crucial for inducing tumor formation [197]. These data suggest that alterations in the gut microenvironment favor the growth of potentially harmful pathogens and pathobionts and that the increase in number enables them to exhibit their capability to induce tumors.

In the following section, we will mainly focus on how single species of the microbiota contribute to arising cancers by becoming pathobionts in the context of dysbiosis.

### 5.1. Escherichia coli.

As part of the natural flora, *E. coli* species are ubiquitously present in the colon of humans and animals [198]. They are facultative anaerobic, Gram-negative, rod-shaped bacteria equipped with motility mediated by peritrichous flagella [199]. Several pathogenic *E. coli* strains, enterohaemorrhagic and enteroadherent *E. coli* (EHEC and EAEC), have evolved mechanisms to actively attach to the epithelium, which will not be addressed here. We will instead focus on commensal *E. coli* strains that reveal pathobiontic activity in the context of carcinogenesis. Among the several subspecies of *E. coli*, *pks*^+^ strains of the phylogroup B2 have come into focus because their pathogenicity island is responsible for colibactin synthesis [200,201]. Colibactin, first described in 2006, is a genotoxin that has been shown to directly bind DNA and cause DSBs via alkylation, leading to activation of the DNA damage response, cell cycle arrest and eventually cell death [201,202]. Colibactin’s two electrophilic cyclopropane residues undergo ring opening, forming covalent cross-links with the DNA to yield an unstable DNA adduct [203,204]. These inter-strand crosslinks may result in replication stress and DSBs that could cause aberrant DNA damage repair (reviewed in Reference [205]). The described cytopathic effects, including megalocytosis, were observed in different mammalian cell lines and depended on the establishment of direct cell-contact between bacteria and host [202]. *E. coli* has been associated with CRC, with a highly increased abundance in tumor samples compared to control biopsies [206]. Subsequently, another research group could show in vivo that the *pks* island is indeed required for generation of DNA damage and promotes carcinogenesis in a colitis-susceptible interleukin-10-deficient mouse model [207]. Interestingly, phylogenic group B2 *E.coli* strains that are able to persist in the gut are significantly more likely to carry the *pks* island compared to short-term or intermediate duration colonizers [208]. Recent epidemiological studies from Sears and colleagues revealed that *pks*^+^ *E. coli* is present more often in CRC patients than in healthy individuals and treatment of cell lines and primary cells with *pks*^+^ clinical isolates leads to syncytia and megalocytosis in these cells [209]. Strikingly, *pks*^+^ strains are also enriched in patients with familial adenomatous polyposis (FAP) pointing out a surprising connection of carcinogenic bacteria with a disease that is considered to hereditarily increase the risk for CRC [210].

With the advent of high-throughput sequencing and advanced genomic analyses, cancer signatures induced by mutagenic substances have been highlighted and they may also help to understand where and how mutations arise during bacterial infections. For *pks*^+^ *E. coli,* whole-genome sequencing of long-term infected organoids as well as samples from CRC patient cohorts showed that they exhibit the same mutational signature–hinting for the first time at a mutational process resulting from exposure to colibactin-producing bacteria [211]. Whole-exome sequencing of a CRC patient cohort even allowed definition of the exact AT-rich sequence motif linked to a specific shape of the DNA that is enriched in the regions in which colibactin promotes DSBs [212]. While this demonstrates that a subset of cancer patients was in contact with colibactin and that colibactin induced mutations in such patients, it is not clear whether indeed these mutations directly contributed to cellular transformation. Indeed, colibactin signatures were also found in healthy subjects. Therefore, more mechanistic studies are required to fully capture the carcinogenic potential of colibactin.

### 5.2. Other Commensals

Colibactin is also expressed by other *Enterobacteriaceae,* such as *Klebsiella*, *Enterobacter* and *Citrobacter* [213] and it will be important to investigate whether such bacteria may also contribute to CRC. Overall, there is increasing evidence for the role of colibactin in CRC development. However, it is likely that only a small proportion of patients that harbor colibactin-producing bacteria will develop malignant disease. This could be due to the fact that under homeostatic conditions such bacteria will not get in direct contact with the epithelium—and especially with epithelial stem cells. Understanding under which conditions such interactions may occur is a critical next step to obtain a full picture of colibactin’s carcinogenic capacity.

Another member of the microbiota, *Fusobacterium* has also been found in tumors, pointing towards a link with cancer (reviewed in Shang and Liu [214] and Zhou, et al. [215]). However, it remains unclear if the presence of these bacteria in tumor samples is a cause or a consequence of the developing tumor. It might well be that instead of promoting the initial transformation, distinct microbes merely find a niche inside the developing tumor tissue and are not etiologically linked with the malignancy. However, in the case of *Fusobacterium,* the bacteria have also been shown to promote the progression of CRC via suppression the immune system and alteration of signaling pathways [216].

## 6. Conclusions

In summary, direct interaction between pathogens or pathobionts and the epithelium is a common feature required for their pro-oncogenic effects. Bacteria must have the ability to make contact with epithelial cells or at least to be in close proximity, for their pro-carcinogenic virulence factors to come into play. Such effects involve both the ability to alter cell signaling as well as to induce genomic injuries directly. While for *H. pylori* a connection with gastric cancer is well accepted, it is becoming increasingly clear that a link between CRC and other bacteria also exists. Particularly for the genotoxin colibactin, which is expressed in different members of the microbiota, a causative role in colorectal carcinogenesis becomes apparent. An interdisciplinary effort involving epidemiological and mechanistic studies is required to dissect the full potential of colibactin and other virulence factors to cause genotoxic damage and to understand how they contribute to human gastrointestinal carcinogenesis. The discovery of mutational signatures has opened up a promising research field. These insights are important as they might pave the way for bacterial eradication approaches to prevent gastrointestinal cancers from developing. As was shown for colibactin-related mutations in cancer patient genomes, mutations in tumor driver genes are more likely to happen in the rectum than in the colon [212], which highlights the need for further research into the spatial colonization preference of bacteria in order to understand which regions are especially prone to mutations and thus the development of tumors. This could allow for more sophisticated screening methods and risk assessments for cancer development.

As mentioned above, many essential metabolites are produced by the microbiota, such as short-chain fatty acids, amino acids and vitamins [45,217,218,219,220]. However, some of these metabolites can exhibit detrimental activity on the epithelium as discussed for butyrate [93]. Metagenome analysis of the human microbiota has revealed that the average person carries more than half a million bacterial genes in their gut, many of which encode for proteins with catalytic activity [221]. As gut microbes are competing within their niche, enzymes and other products of metabolic pathways might lead to a competitive advantage for certain species and additionally damage host cells. Both are the case for the genotoxic DNase I homolog CDT [179,182]. Therefore, it would be of great interest to further investigate these and other data sets to discover new enzymes and metabolites linked to cancer onset. Emerging new techniques in the field of proteomics and metabolomics will pave the way for a quantitative assessment of how these molecules act on a functional level.

Knowledge about the exact mechanisms by which mutations are induced directly by bacteria and how these mutations persist in the epithelium after the acute infection event has passed, will help us to understand the transformational processes occurring in the GI tract. To achieve this, research should focus on a detailed understanding of which cell types are targets for mutations and how these cells benefit from the introduced genetic alterations in terms of their fitness to survive and to repopulate the epithelium. Sophisticated model systems will be needed in order to mimic the complex processes of tissue homeostasis and renewal while challenging cells with the mentioned virulence factors that contribute to GI carcinogenesis.

In addition to basic research, these data should be put into clinical context. In order to increase the medical relevance, further evidence is needed to convince the biomedical community that certain bacteria can drive carcinogenesis. This also calls for strategies to prevent bacteria-induced malignancies, for example, through microbial eradication strategies or by preventing such bacterial infections in the first place. If such therapeutic approaches prove successful, they will raise awareness of the relationship between bacteria and cancer and generate increased financial support for accelerated research in this area.

## Supplementary Materials

The following figures are available online at https://www.mdpi.com/1422-0067/21/20/7439/s1. Table S1: Overview of relevant reviews.
