# Supplementary material for: Microbe-Driven Genotoxicity in Gastrointestinal Carcinogenesis
Source: Int J Mol Sci. 2020 Oct 9;21(20):7439. doi: 10.3390/ijms21207439 (PMC7587957; doi:10.3390/ijms21207439)
Supplement: Supplementary file 1 [file ijms-21-07439-s001.pdf]

Table S1. Overview of relevant reviews.

Ref.	Authors	Title	Journal/Source	Year
[1]	Chang, C.-S.; Kao, C.-Y.	Current understanding of the gut microbiota shaping mechanisms.	Journal of Biomedical Science	2019
[2]	Vereecke, L.; Beyaert, R.; van Loo, G.	Enterocyte death and intestinal barrier maintenance in homeostasis and disease.	Trends in Molecular Medicine	2011
[3]	Chumduri, C.; Gurumurthy, R.K.; Zietlow, R.; Meyer, T.F.	Subversion of host genome integrity by bacterial pathogens.	Nature Reviews Molecular Cell Biology	2016
[4]	Frick, J.S.; Autenrieth, I.B.	The gut microflora and its variety of roles in health and disease.	Current Topics in Microbiology and Immunology	2013
[5]	Di Domenico, E.G.; Cavallo, I.; Pontone, M.; Toma, L.; Ensoli, F.	Biofilm Producing Salmonella Typhi: Chronic Colonization and Development of Gallbladder Cancer.	International Journal of Molecular Sciences	2017
[6]	Ducarmon, Q.R.; Zwittink, R.D.; Hornung, B.V.H.; van Schaik, W.; Young, V.B.; Kuijper, E.J.	Gut Microbiota and Colonization Resistance against Bacterial Enteric Infection.	Microbiology and Molecular Biology Reviews	2019
[7]	Young, K.T.; Davis, L.M.; DiRita, V.J.	Campylobacter jejuni: molecular biology and pathogenesis.	Nature Reviews Microbiology	2007
[8]	Rajagopala, S.V.; Vashee, S.; Oldfield, L.M.; Suzuki, Y.; Venter, J.C.; Telenti, A.; Nelson, K.E.	The Human Microbiome and Cancer.	Cancer Prevention Research (Phila)	2017
[9]	Thakur, B.K.; Malaisé, Y.; Martin, A.	Unveiling the Mutational Mechanism of the Bacterial Genotoxin Colibactin in Colorectal Cancer.	Molecular Cell	2019
[10]	Shang, F.-M.; Liu, H.-L.	Fusobacterium nucleatum and colorectal cancer: A review.	World Journal of Gastrointestinal Oncology	2018

1. Chang, C.-S.; Kao, C.-Y. Current understanding of the gut microbiota shaping mechanisms. *J. Biomed. Sci.* **2019**, *26*, 59, doi:10.1186/s12929-019-0554-5.
2. Vereecke, L.; Beyaert, R.; van Loo, G. Enterocyte death and intestinal barrier maintenance in homeostasis and disease. *Trends Mol. Med.* **2011**, *17*, 584–593, doi:10.1016/j.molmed.2011.05.011.
3. Chumduri, C.; Gurumurthy, R.K.; Zietlow, R.; Meyer, T.F. Subversion of host genome integrity by bacterial pathogens. *Nat. Rev. Mol. Cell Biol.* **2016**, *17*, 659–673, doi:10.1038/nrm.2016.100.
4. Frick, J.S.; Autenrieth, I.B. The gut microflora and its variety of roles in health and disease. In *between Pathogenicity and Commensalism*; Springer: Berlin, Heidelberg, Germany, 2012; pp. 273–289.
5. Di Domenico, E.G.; Cavallo, I.; Pontone, M.; Toma, L.; Ensoli, F. Biofilm Producing Salmonella Typhi: Chronic Colonization and Development of Gallbladder Cancer. *Int. J. Mol. Sci.* **2017**, *18*, doi:10.3390/ijms18091887.
6. Ducarmon, Q.R.; Zwittink, R.D.; Hornung, B.V.H.; van Schaik, W.; Young, V.B.; Kuijper, E.J. Gut Microbiota and Colonization Resistance against Bacterial Enteric Infection. *Microbiol. Mol. Biol. Rev.* **2019**, *83*, e00007–e00019, doi:10.1128/MMBR.00007-19.

7. Young, K.T.; Davis, L.M.; DiRita, V.J. Campylobacter jejuni: molecular biology and pathogenesis. *Nat. Rev. Microbiol.* **2007**, *5*, 665–679, doi:10.1038/nrmicro1718.
8. Rajagopala, S.V.; Vashee, S.; Oldfield, L.M.; Suzuki, Y.; Venter, J.C.; Telenti, A.; Nelson, K.E. The Human Microbiome and Cancer. *Cancer Prev. Res.* **2017**, *10*, 226–234, doi:10.1158/1940-6207.CAPR-16-0249.
9. Thakur, B.K.; Malaisé, Y.; Martin, A. Unveiling the Mutational Mechanism of the Bacterial Genotoxin Colibactin in Colorectal Cancer. *Mol. Cell* **2019**, *74*, 227–229, doi:10.1016/j.molcel.2019.04.007.
10. Shang, F.-M.; Liu, H.-L. Fusobacterium nucleatum and colorectal cancer: A review. *World J. Gastrointest. Oncol.* **2018**, *10*, 71–81, doi:10.4251/wjgo.v10.i3.71.
